# The coupling of pathways and processes through shared components

**DOI:** 10.1186/1752-0509-5-103

**Published:** 2011-06-29

**Authors:** Daniel D Seaton, J Krishnan

**Affiliations:** 1 Dept. of Chemical Engineering and Centre for Process Systems Engineering Imperial College London, South Kensington Campus, London, SW7 2AZ, UK; 2 Institute for Systems and Synthetic Biology Imperial College London, South Kensington Campus, London, SW7 2AZ, UK

## Abstract

**Background:**

The coupling of pathways and processes through shared components is being increasingly recognised as a common theme which occurs in many cell signalling contexts, in which it plays highly non-trivial roles.

**Results:**

In this paper we develop a basic modelling and systems framework in a general setting for understanding the coupling of processes and pathways through shared components. Our modelling framework starts with the interaction of two components with a common third component and includes production and degradation of all these components. We analyze the signal processing in our model to elucidate different aspects of the coupling. We show how different kinds of responses, including "ultrasensitive" and adaptive responses, may occur in this setting. We then build on the basic model structure and examine the effects of additional control regulation, switch-like signal processing, and spatial signalling. In the process, we identify a way in which allosteric regulation may contribute to signalling specificity, and how competitive effects may allow an enzyme to robustly coordinate and time the activation of parallel pathways.

**Conclusions:**

We have developed and analyzed a common systems platform for examining the effects of coupling of processes through shared components. This can be the basis for subsequent expansion and understanding the many biologically observed variations on this common theme.

## Background

Intracellular signalling networks are characterised by their ability to perceive and integrate a variety of signals in order to make decisions. In order to do this, their components often interact with multiple entities at multiple locations, allowing them to receive and send multiple signals. This property is seen, for example, in proteins capable of multiple allosteric interactions such as n-WASP [1], WAVE [2], Cdk-2 [3], and PLC [4]. There are also many examples of enzymes capable of modifying multiple substrates [5-9], including signalling proteins such as cyclin-dependent kinases [10,11], and ubiquitin ligases [12]. Similarly, substrates may be modified by multiple enzymes, as is the case for the p53 tumour suppressor [13] and many GTPases. Each of these reactions may take place while bound to various adaptor and scaffold structures, as is common for instance in MAPK cascades [14]. Finally, all of these interactions and reactions may take place in diverse cellular locations, with many proteins having been identified as having multiple subcellular localizations [15]. Commonly known examples of this are cell-cycle proteins such as cyclins, which shuttle between the nucleus and cytoplasm, and a wide variety of membrane-binding signalling proteins, which may also be present in the cytoplasm. The sharing of components between pathways and locations is widespread and one of the most basic ways in which processes may be coupled.

A notable aspect of signalling in biological systems, and one which distinguishes them from many engineered systems, is that it is inherently bidirectional. Whenever a signal is being sent or received, components must interact with one another, and/or change location, and are occupied by those actions for finite periods of time. Therefore a signal is itself modified when it is perceived by a downstream signalling element. The extent of bidirectional signalling has been termed retroactivity [16]. While retroactivity may be low in some cases, and while there may be reasons for systems minimising it in some cases, it is nonetheless likely to have a non-trivial effect in other cases. This is particularly true, and especially significant, in networks containing elements with multiple interactions. Signalling networks involve many proteins with multiple interactions and multispecific enzymes, where many of the elements are similar in concentration. Therefore, proteins may be shared between multiple pathways, and the question arises as to what functional roles these multiple interactions and consequent bidirectionality might play in cellular signal processing in biological systems.

The most important aspect of signalling networks which may be affected by multiple interactions is their ability to perceive and integrate signals, and thereby perform logical operations. Multiple interactions and bidirectional signalling may affect the input-output response of pathways, and may be particularly relevant to investigating signalling crosstalk [17-19]. Crosstalk occurs when multiple pathways share components. Despite this coupling, signalling networks are often seen to allow one input to specifically regulate only one or a few outputs. This is termed signalling specificity. Likewise, it is observed that in some networks particular outputs are regulated by only one or a few inputs, termed signalling fidelity. It is important to understand the role of crosstalk in such networks and how signalling specificity and fidelity may be maintained. Another phenomenon observed in signalling is the temporal coordination of processes with one another. Examples include events such as mitosis [20] and the assembly of large protein complexes involved in flagellar motors [21]. Bidirectional aspects of signalling may affect or even contribute to these properties in a very non-trivial manner, and therefore are of direct biological relevance. Overall our studies provide important insights which help bridge descriptions of networks at local and global levels.

Further to the biological implications discussed above, there are important implications for the ways in which biological signalling circuits are modelled. Mathematical modelling has been used to analyse and understand many signalling networks. Such models frequently consider enzyme-substrate complexes only implicitly, often using Michaelis-Menten kinetics or other simplifications such as the quasi-steady state assumption (QSSA) [22-24]. It has been recognised that these simplifications may have significant effects on the behaviour of models, as seen recently in analyses of ultrasensitive and multistable reaction networks [25-27]. Another implication is that modular decompositions of networks, which may allow rapid simulation and more straightforward analysis, must be undertaken with care. The analysis which we present is relevant to both these aspects.

In order to focus on the essential aspects of coupling of processes through shared components, and hence provide insights into the various issues mentioned above, we develop an appropriate modelling/systems framework. The modelling framework incorporates two components which bind exclusively to a common third component, and are therefore indirectly affected by each other. The model incorporates the production and degradation of all components, thus allowing each component to serve as a "signalling port". Having developed the basic model, we proceed to systematically examine the signal processing through this module, as this sheds direct light on the above issues. It is worth emphasizing that in this minimal general setting, systems analysis provides transparent and important insights which are relevant to a wide range of systems/contexts where the above feature(s) occur. We further build on the study of the basic model to include additional features such as spatial diffusion/localization, and other complexities in signal propagation such as threshold effects. Throughout, we focus on the effects of coupling signalling elements through shared components, revealing different facets of such generic coupling.

This paper is organized as follows. In the next section, we present the basic modelling framework which we employ. Following this, we systematically examine the steady state and temporal signal processing in this module in turn. We illustrate the relevance of the analysis in specific biological contexts. We then examine the effect of the additional elements mentioned above. Finally we conclude with a synthesis and discuss additional applications and extensions.

## Methods

### The basic model of coupling of signalling pathways

Here, we develop a basic model of pathway coupling - the sharing of one component between processes. In its most basic form, the coupling of processes and signalling can be studied via a simplified ordinary differential equation model, which involves the interaction of three species A, B and X. A and B each bind exclusively to X, and thus X serves as a factor which couples the dynamics of A and B. We formulate the model in a general form, so that the essential insights can be extracted in a transparent and generalisable way. Details of additional models used and the parameter values used in simulations are available in Additional File 1.

We begin by modelling the interaction of A and X alone. The processes which are modelled are the binding of A and X to produce a complex AX, the dissociation of the complex, and the independent production and degradation of A and X. The dynamics of this system are governed by the equations:(1)

Here, [X], [A], and [AX] denote the concentrations of each species A, X and the complex AX. In the above equation, k_a1 _and k_a2 _denote the binding and unbinding rate constants, k_px_, k_pa _denote the production rates of the species X and A respectively, and k_dx_, k_da _denote the degradation rates of these species.

This model is, in general, non-trivial to solve analytically, although this may be facilitated if the degradation rates of all species are equal [28]. There are, however, several reasonable simplifications which allow some initial analysis to be performed. First of all, if production and degradation of species may be assumed to occur on a longer timescale than complex formation, then these terms may be neglected and this results in the equations (in dimensionless form):(2)

Note that in these simplified equations, the total amounts of A and X are conserved, and hence information about the availability of these species is contained in the initial conditions. These expressions may be condensed by applying conservation conditions using the total quantities of A, and X (denoted [A_T_] and [X_T_], respectively).

**Figure 1:**
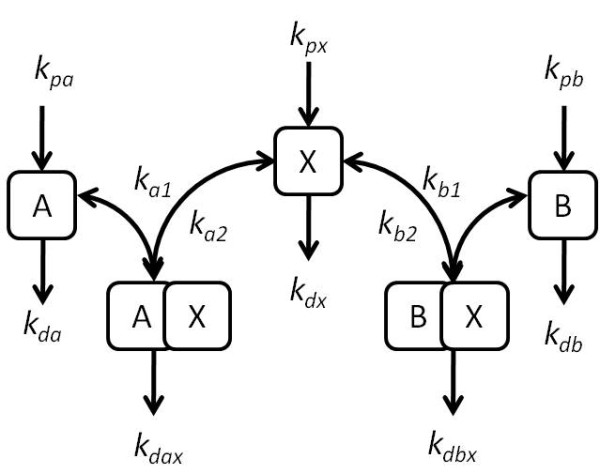
**Basic model schematic**. A schematic of the basic model is shown here. The species X interacts with both A and B. All species undergo constant production and degradation.

All we have done up to this point is describe the dynamics of a protein, X, involved in one process, A, as has been modelled previously [28]. However, we are primarily interested in what happens when X is involved in more than one process, since it is these cases in which the coupling comes into play. Therefore, we introduce a second process, B, and can make use of the same model to describe its interactions with X (see Figure 1):(3)

The above model incorporates the binding of B to X to form a complex BX, as well as the dissociation, and in addition includes the production and degradation of B (rate constants k_b1_,k_b2_,k_pb_, and k_db _respectively). While we have described the production and degradation of species, we stress that this need not be taken as protein synthesis and degradation - it includes, for example, the rate of formation of a particular post-translationally modified form of a protein. This is significant because these processes may occur on a much faster timescale than protein synthesis and degradation.

Some of the analysis will be concerned with the steady-state of these models. In this, the equilibrium constants for the complex formation of A and B with X (the ratio of binding to dissociation rate constants) become relevant parameters of interest. We denote these *K_A _*(= *k_a_*_1_/*k_a_*_2_) and *K_B _*(= *k_b_*_1_/*k_b_*_2_).

### Variation of inputs and outputs

We note at the outset that the model is a general model of components A and B, interacting through competitive binding with the element X. This model allows modulation of the levels of each of these components by external signals through their rates of production and degradation. Throughout the paper, we are primarily interested in two essentially different ways in which the levels of components are modulated by external signals. In the first case, we examine how changes in production of the shared component, X, are propagated to affect the levels of free A and B, and the levels of the complexes AX and BX, and therefore modify both pathways in which X participates. In the second case, we examine how changes in the production of the components A and B affect the levels of all components and complexes. This corresponds to the pathways being controlled while the shared component remains constant. Through this analysis, we hope to understand the range of behaviours available to such systems, and their possible biological significance. In particular, we examine how shared components may coordinate processes, and how processes may remain independent despite sharing components.

At this stage we make very few assumptions about the nature of the downstream processes involving the complexes AX and BX. Later in the paper, we build on the existing modelling framework to examine certain additional features in the downstream processes from our perspective.

Our results involve analyzing the models using simulations (performed in MATLAB using ode15 s) and analytical results. We choose a representative set of parameters, and examine the effect of the change in important parameters as appropriate.

## Results and discussion

The results are organized as follows: we use our modelling framework to study how the system responds to different signals from both steady state and temporal perspectives. We then build on our analysis to examine a number of biologically motivated variations to our structure, which include additional components, downstream switching elements and spatial signal transduction, and discuss their possible biological significance. We start by examining the case where a signal modulates the production of the shared component, X, and continue by examining the case where signals modulate the components A and B, both separately and simultaneously.

### Modulation of the shared component

We begin by analysing the steady state response of the system to changes in the production of X. From the perspective of signal propagation, this may be regarded as signal processing through "diverging pathways”. Assuming that the rates of degradation of all components are equal, we can write the total quantity of each component in terms of the production and degradation rates ([*X_T_*] = *k_px_/_kd_*, [*A_T_*] = *k_pa_/k_d _*[*B_T_*] = *k_pb_/k_d_*, where [*X_T_*], [*A_T_*], and [*B_T_*] refer to the total concentrations of X, A, and B, respectively). This allows us to analyse the model in terms of its response to [*X_T_*], allowing more transparent explanation of the results.

**Figure 2:**
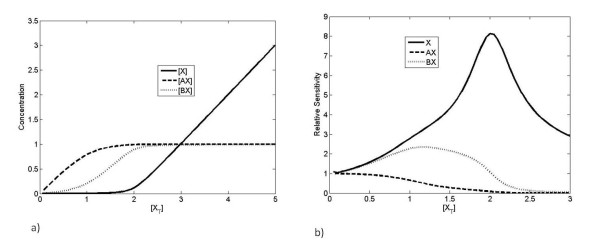
**Steady-state response to changes in production of X**. a) The pattern of linear and saturating responses to changes in [*X_T_*] is shown for the case *K_A _*>>*K_B _*>> 1. b) The relative sensitivity (see Appendix for definition and analysis) of each component with respect to [*X_T_*] is plotted showing how it peaks above one for free X and BX, but decreases to zero for AX.

Figure 2 shows the response of the system when A and B are produced and degraded at equal rates, for the case where X binds more strongly to A than to B. We note that there are essentially three regimes of response. In the first regime, all processes are unsaturated and X is mostly taken up by A, since *K*_*A *_≫ *K*_*B*_. In the second regime, process A has become saturated, and X is taken up by B. In the third regime, both processes have become saturated and X accumulates in its free form. These regimes show an "ultrasensitive" response of BX and free X, where a threshold in the total amount of X present must be reached before a significant response is observed. For our purposes, it is sufficient to think of "ultrasensitivity" as an effect involving increased absolute and relative sensitivity, along with a concomitant threshold effect (see Appendix for details, and see [26] for a discussion of technical definitions of "ultrasensitivity").

Some basic analysis provides direct quantitative insight (see appendix for a more detailed analysis of the response of the system to changes in total X). For simplicity, the analysis is performed for the case where the production and degradation of X, A and B are neglected. In this case, the behaviour of the system is monitored for the case where an addition of free X (and hence total X) is imposed at t = 0. An inspection of the steady state equations reveals that the concentration of the complexes is proportional to the total amount of X, [*X_T_*]. At steady state, each of the complex formation/dissociation reactions is at equilibrium:(4)

At steady state and using the conservation condition [*X_T_*] = [*X*] + [*AX*] + [*BX*], we get the following equations for the response of AX, BX and free X to changes in the total concentration of X:(5)

Note that [A] and [B] are the concentrations of free A and free B and hence implicitly depend on the total X in the system. Assuming that the binding affinity of A is much greater than that of B, we can discern three regimes in the response. These three regimes can be described in terms of the saturation of A and B. Initially, since the binding affinity of A is much greater than B and A and B are present in equal amounts *K_A_*[*A*] + *K_B_*[*B*] + 1 ≈ *K_A_*[*A*], and most of the available X forms complexes with A (this implicitly assumes that the available A and B is in excess of X). Once A is depleted, the quantity *K_A_*[*A*] becomes dwarfed by *K_B_*[*B*], and *K_B_*[*B*] + *K_B_*[*B*] + 1 ≈ *K_B_*[*B*], and most of the available X forms complexes with B. This is what underlies an "ultrasensitive" response in BX as the total concentration of X is increased. This parallels the effects discussed by Buchler et al [28,29], although we note that the relative sensitivity (the sensitivity scaled by concentrations, see appendix for details) in the complex BX is less than the relative sensitivity observed in the free X (see Figure 2). Once B is depleted, all remaining X is added to the free pool. We note that the "ultrasensitivity" in response of the B pathway depends on suppression of signal at low values of the input (in this case [*X_T_*]) by the A pathway. This requires that *K_A_*[*A*] ≫ *K_B_*[*B*], which is a condition on relative affinities rather than absolute affinities. However, the absolute sensitivity in [*BX*] also depends upon a high linear response once A is depleted and that suppression is overcome, requiring *K_B_*[*B*] ≫ 1. Therefore, the response observed requires the system to satisfy the condition *K_A_*[*A*] ≫ *K_B_*[*B*] ≫ 1. The results are illustrated in Figure 2.

Other classes of regime may similarly be discerned, depending on the relative amounts of A and B initially and the affinities. For instance suppose K_A_[A] >> 1 >> K_B_[B], then we see that as X_T _is increases, A is largely taken up, but as A depletes, much of the extra X remains free rather than bound to B. The other case where 1 >> K_A_[A] >> K_B_[B] throughout is one where most of the X is unbound, and is hence of less interest.

Returning to the above analysis for the case when *K_A_*[*A*] ≫ *K_B_*[*B*] ≫ 1, we note that our analysis and conclusions were based on a steady state analysis and the factors which enter the analysis are the equilibrium constants. We now examine how such a network responds to temporal signals.

In order to do this, we take the full system at steady state and change the production rate of X at t = 0. Note that a slow change in the quantity of X would leave the system in a quasi-steady state, and the results would follow directly from the steady state analysis presented above. Thus, we examine cases where rapid changes in the quantity of X available are induced.

**Figure 3:**
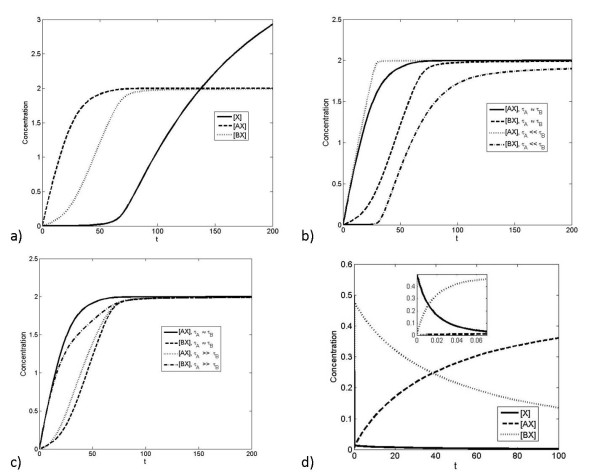
**Dynamic response to step-change in production of X**. a). This figure shows how a step change in production rate of X (starting from 0) imposed at t = 0, affects the concentration of all different components. Here, *K_A _*>>*K_B _*>> 1. Note that eventually the concentration of free X also reaches a steady state. b) When the timescale of the lower-affinity interaction (interaction of X with B) is lengthened, the higher-affinity interaction saturates at earlier times, and the lower-affinity interaction saturates at later times. c) When the timescale of the higher-affinity interaction (interaction of X with A) is lengthened, the order in which the complexes are formed can be reversed, so that the lower-affinity interaction saturates at an earlier time than the higher-affinity interaction. Note that in this case the total amount of X in the system finally is clearly greater than the total of A and B put together, so that eventually, most of A and B are present in complexed form. (d) The case where the lower affinity component is faster is shown. Here a sudden change in production of X is imposed so that the total X does not exceed the total amount of A and B. Here we see that at early times (see inset) B takes up most of the X, while at longer times, the concentration of the complex BX gradually decreases and that of AX eventually increases.

If we apply a step increase in the production of X, we find that A responds first, followed by B as discussed above - this is illustrated in Figure 3. This is for the case where the relative affinity of A is greater than that of B and where the timescales of the pathway A are much faster than that of B. In this case the dynamic signal processing in this circuit essentially mirrors the steady state signalling and response discussed above. This is further consolidated by analytical results (see Appendix). We further note that, when X is present at low levels, an increase in the production of X will primarily affect A, with little dynamic response in B.

Also shown in Figure 3 is that, in the opposite case, if the relative time scales of the high affinity binding/unbinding are changed (keeping the equilibrium constant fixed) it is possible to saturate the low-affinity component, B, more rapidly than the high-affinity component. In Figure 3, a case where a sufficiently high change in X is considered so that both A and B are essentially saturated at steady state. For intermediate levels of X, what can be observed is that the low affinity component complex is rapidly formed before a gradual redistribution of X between the pathways. Thus if the low affinity component B is the faster responding component, then a step change in X (in this range) will affect B first, before it gradually reduces with the X "leaking" back to the A pathway. Thus in this regime, a step change in X results in a marked but essentially transient response for BX, and a much more gradual response for AX. Thus BX displays a faster but adaptive response, while AX displays a slower but persistent response. We further note that if the total X is increased past a level which ensures saturation of the component A, then BX displays a response which is partially adaptive. This partial adaptation (underadaptation) can be traced to the saturation of the "inhibitory" pathway A.

This can be further understood by analytical studies (see Appendix). This illustrates the importance of kinetics in addition to steady state and quasi-steady state analysis in understanding the temporal order of activation of the pathways.

**Figure 4:**
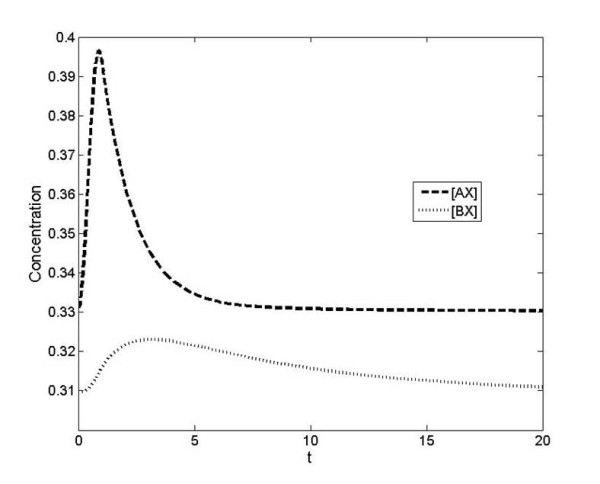
**Dynamic response to pulsatile change in production of X**. The response to a rectangular pulse of X imposed starting at t = 0 is considered here. Here the affinities of the interactions are equal (*K_A _*= *K_B _*= 1), but the timescales are different (*τ_A _<< τ_B_*). The faster interaction (with A) takes up X more quickly, but also releases it more quickly.

To further examine the temporal response, we input a square pulse in the production of X. The response is seen in Figure 4, where the faster of the two pathways responds quickly, but also recovers more quickly. In contrast, the slower pathway registers a more prolonged but shallower response. Again, the insights from the numerical simulations can be complemented by analytical studies.

Overall, the analysis above provides insight into how the coupled pathways process steady and temporal signals through their shared component and propagate them downstream, and the role of other factors in modulating this process.

### Modulation of each pathway alone, and together

We now use our modelling framework to examine the case where input signals "converge" on common target. This is done in our model by changing the production of A and B, and keeping the production of X fixed.

**Figure 5:**
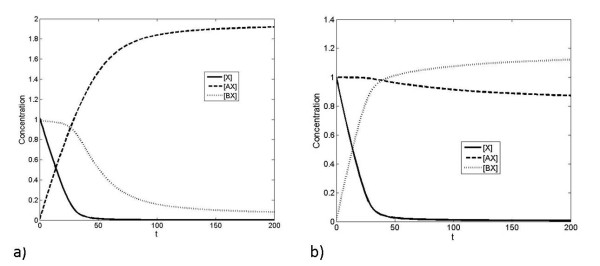
**Dynamic response to change in production of A or B alone**. The response to step change in production of A (starting from zero) is shown in a), with *K_A _*>>*K_B _*>> 1. The steady state analysis shows that this should result in a depletion of BX and X. The temporal analysis shows the expected ordering of this depletion: first X is depleted, then BX. Note that the initial conditions correspond to a steady state with a non-zero production rate of B. The response of the system to a step change in production of B (starting from zero) is shown in b). The initial conditions here correspond to a steady state with a non-zero production of A. The result is qualitatively similar, except production of B is less able to deplete AX, since *K *>>*K_B_*.

We first consider the case when the input is applied only to A (the high affinity component). Figure 5 shows the result wherein the concentration of the complex AX increases and the concentration of free X decreases followed by that of the complex BX. This shows how signalling through one of two "converging pathways" may affect the second one. Also shown is the case where the step input is applied to the low affinity component. Here BX builds up, depleting X, and then AX, but overall the AX concentration doesn't decrease as substantially as before simply because the low affinity component isn't able to outcompete the high affinity component as effectively. Thus here the retroactivity effect of pathway B on pathway A is weak, when compared to that of pathway A on B.

**Figure 6:**
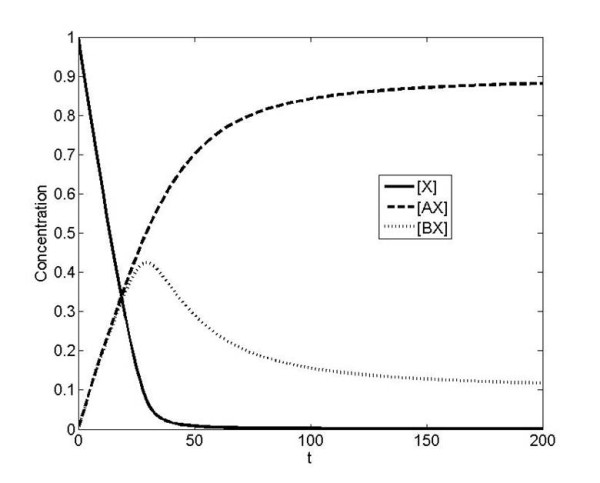
**Dynamic response to change in production of A and B together**. The response to a simultaneous step change in production of A and B (both starting from zero) is shown, where *K_A _*>>*K_B _*>> 1. We observe that both [*AX*] and [*BX*] rise initially. However, as [*X*] decreases competition between A and B becomes significant, and the higher affinity interaction of X with A means that it outcompetes B at later times.

Now we consider the effect of simultaneous step changes provided to A and B (Figure 6). We find that the concentrations of both complexes initially rise, but that eventually the concentration of the complex of the low affinity component is depleted by the retroactivity effect. We thus see how a step change in production and A and B together results in a partially adapting response of BX, purely due to competing effects.

The above simulation has relevance for the case of pathways which are costimulated, and which have common downstream targets. Again, in the above case, the simulation studies can be complemented by analytical studies which distil the effects seen above. This is discussed in the Appendix.

We briefly discuss analytical results from a simplified perspective, to complement those in the previous section. We again consider a setting where the affinity of A for X is very high and much greater than that of B (*K_A _*>>*K_B_*). For specificity, we assume that under basal conditions, an equal amount of A and B is present. Suppose the production and degradation of all components is ignored, then we have conservation of total A, B and X as discussed in the previous section. Further the fraction of X in a complex with A and B at steady state is in fact given exactly by the expressions in the previous subsection. Now suppose we consider a case, where extra A and B are introduced in equal amounts at t = 0, and the system is allowed to evolve. The response of the system depends on the affinity of B. If B also has sufficiently high affinity for X then initially both A and B are present almost completely as complexes (assuming sufficient X is available). Now when A and B are added, at steady state if enough X is available, they will both be taken up as complex. Therefore from the point of the individual pathways, we see an essentially proportional response. On the other hand if the total amount of A and B in the system exceeds that of X, then at steady state, it is A which is present entirely in complex form, while B is bound to the X which is not taken up by A. Thus here while the A pathway shows output directly reflects the addition of A, the B pathway in fact reflects only a partial complexing, which in turn directly depends on the presence of A. At this stage, any further (concurrent) additions of A and B increase the AX concentration, but decrease the BX concentration. As the level of the input increases, the AX concentration increases till it essentially equals the total X concentration while the BX concentration reduces till it becomes zero.

Similar trends also hold good if the affinity of B for X is at intermediate levels. Here, only a fraction of B is found bound to X and this depends also on the amount of B in the system. If A and B are added, when A and B are at relatively low levels, this results in the complete uptake of A and an increase in complex of B, due to the presence of increased B in the system. However, when the levels of A and B are further increased, the concentration of AX increases, and the concentration of BX decreases, because the effect of additional B is counteracted by the fact that less X is available for binding to B.

Again, we can go beyond steady state responses to examine kinetic effects. We see that if A is the faster responding pathway, then the insights of the steady state analysis are mirrored in the way the concentrations of the complexes AX and BX change. When available X is present, the added A binds to available X, followed by B if some X remains. When the levels of A and B combined exceed that of X, and unbound A exists, the complex BX gradually unbinds to allow for the subsequent rapid binding of A with released X. Thus the kinetics of the complex BX dissociating may limit the rate at which free A is subsequently absorbed (and likewise the concentration of BX is reduced due to this effect). Hence one may observe that the increase of AX occurs in two phases, a fast phase where it binds with free X and a slow phase where it relies on dissociation of BX.

We can also examine the opposite case, where B is the faster component. Here the effect of B on X is faster, but this is gradually eroded by the extra A. When A and B levels are such that their total is less than that of X, we see an increase of BX followed by that of AX. When in a stimulus A and B levels exceed that of X, we see an increase of BX first followed by a gradual unbinding and increase of AX. Depending on the initial concentrations of A and B and the extent of the stimulus, the concentration of BX may temporally increase before decreasing, and end up either above or even below prestimulus levels. These are qualitatively reminiscent of underadaptive and overadaptive responses respectively. Thus overall we see that depending on the basal level and the strength of the added stimulus, the response of the B pathway may be non-adaptive, partially adaptive, or overadaptive, and this behaviour is determined by the coupling to the pathway A.

We have examined a variety of ways in which this basic system may be modulated by a external signals, both at steady state and dynamically (see Table 1 for a summary). We now build on this to examine how the basic network structure and dynamics may be affected by other additional elements or features. We begin by examining how an additional component can affect the system by forming a complex with A and X.

**Table 1:** Summary of results for the basic model

Scenario	Result
Changing the rate production of the shared Component, X.	At steady state, "ultrasensitivity" may be observed in the response of the lower affinity component, and of free X. When one examines the dynamics of the response of the two pathways, the relative timescales of the interactions are important. Depending on this, the dynamics may mirror the steady state response; alternatively, an adaptive response may be observed in the low affinity component.

Changing the rate of production of A and B.	The two pathways may inhibit one another, with the high affinity component having a greater inhibitory effect. Depending on the timescales of the interactions, the dynamics may either mirror the steady state response, or result in an adaptive response in the lower affinity pathway.

We consider the case where the shared component, X, binds with high affinity to component A, forming the complex AX, and with low affinity to component B, forming the complex BX.

### Combinatorial signalling and the influence of complex formation mechanisms and allostery

It was shown above that, in essence, processes which share a component may act to inhibit one another. The above analysis of the mass-action model applies to situations where A and B form complexes with X by simple independent interactions. In many cases a protein may bind cooperatively, and it may be affected by further binding proteins. In this section we show how such mechanisms influence the potential interaction and coupling between processes. We then suggest a role for this effect in insulating pathways against crosstalk. In particular, since these mechanisms allow single proteins to behave as "AND" gates - active only when receiving both input signals - they allow tuning of specificity through combinatorial signalling.

**Figure 7:**
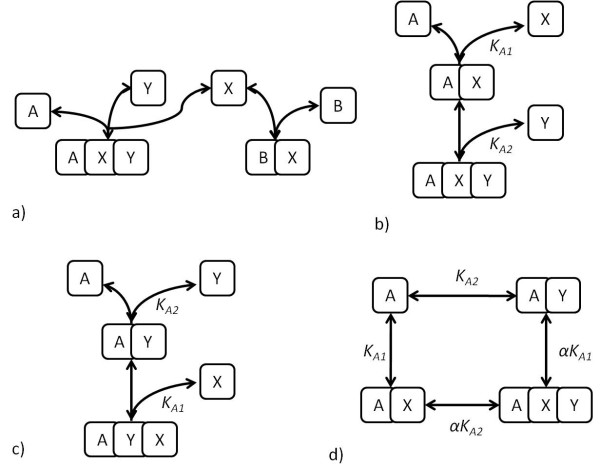
**Schematic of combinatorial signaling**. A situation where an extra player Y is involved in regulating/interacting with pathway X is considered. The signals X and Y combine to form an active complex with A, but X also participates in a complex with B, as shown in a). This involves the formation of a tertiary complex, AXY. Three distinct mechanisms for the formation of this complex are illustrated in b)-d). In b), A binding to X precedes its binding to Y. In c), A binding to Y precedes its binding to X. In d) X and Y bind to A cooperatively, stabilizing it in its active conformation.

In this section, we will consider X as an input affecting A and B; the only difference is that we will have an additional input Y which affects the activation of the A pathway by X. This can occur in different ways: for instance Y can bind with A before it is targeted by X. Alternatively Y can bind with the complex AX only. A third way is if X and Y co-operatively interact with A. Schematic diagrams of all these cases are shown in Figure 7. All of these cases represent a modification of one of the coupled pathways by an extra element Y. Having understood the behaviour of the simpler model previously we can examine what the role of the extra element Y is in coupling the two pathways. Here, we consider the X and Y to be two inputs, with the complexes AXY and BX the outputs.

**Figure 8:**
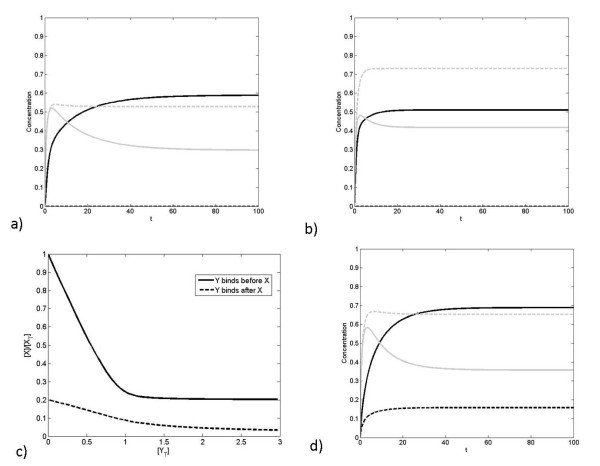
**Combinatorial signalling and specificity**. Combinatorial signalling can increase signalling specificity through competitive inhibition of parallel pathways. This figure depicts the outputs of the A and B pathways when subject (at t = 0) to a change in production rate of X, starting from zero. The concentrations of the active output of pathways A and B are given by black and grey curves, respectively. Solid curves represent the response to both X and Y together, while dashed curves represent the response to X alone. a) shows the response when X binding to A precedes Y binding, b) shows the response when Y binding to A precedes X binding, c) shows how the amount of available Y affects the fraction of free X and d) shows the response when X and Y bind to A cooperatively. In all cases, it is seen that the presence of X and Y together not only activates the A pathway but also inhibits the B pathway, and thus leads to improved signalling specificity. In (a) and (b), the absence of Y leads to zero output from the A pathway. While the qualitative results from (a) and (b) are similar, we some differences in how the total availability of Y affects the results. In the case where Y binds to A before X binds to it, we see that a low availability of Y substantially increases the fraction of free X. This is not the case when Y binds to A after X is bound to it.

As a simple example of the effect complex formation mechanisms can have, consider that the binding of X to A precedes binding by Y (Figure 7b). We denote this, the sequential-binding model of complex formation. A good example of such a mechanism is the binding of substrate to CDK-cyclin complexes, where the cyclin must bind to CDK before the substrate [30]. In this case, two unbinding events must occur before X is free. Y, which corresponds to the CDK-cyclin substrate, is effectively locking in X, which corresponds to the CDK (which may bind to many different cyclins, corresponding to A and B). An analysis of this network reveals the highly non-trivial impact which Y has: this will impact on both the steady state and dynamic behaviour as seen in Figure 8. What is observed is that without Y, the signalling pathway involving A is inactive, and in the presence of Y it is active. Further, in the presence of Y, not only is the A pathway activated but the B pathway is inhibited. In our modelling framework, the introduction of Y can be seen as effectively reducing the dissociation rate of X from the complex AX, modulating the response by further suppressing BX formation at low concentrations of X.

Thus, such a complex formation mechanism can act to increase signalling specificity by effectively combining the cross inhibition and combinatorial signalling methods of reducing crosstalk. In the case of CDK-cyclin complexes mentioned above, given that different cyclins result in different substrate specificities [10], sequestration provides a mechanism for the presence of substrates for a particular cyclin-CDK complex to favour the formation of that complex rather than other cyclin-CDK complexes. Further, deletion of a cyclin may lead to compensation effects from the binding of substitute cyclins, as many are present at significant levels at the same time [31].

The other case, where binding of Y to A precedes binding of X to form the active complex AXY, can also be considered within our framework (Figure 7c). In this case, addition of Y allows binding of X, and so performs the same role in modulating B as A alone did in the basic model. Figure 8 shows again that the presence of Y, increases specificity of A and leads to greater inhibition of B. On the other hand, we see that the presence of Y acts to insulate A from the crosstalk through B via the common connection X. Analytical studies of the above two cases are performed in the Appendix. While in both cases an increased Y acts to inhibit BX formation at steady state, there are some differences. In one case (Y binding with A before binding with X) we see that the amount of Y limits the amount of X involved in this pathway. Thus a low level of Y will substantially reduce the amount of X involved in this pathway. This is not the case for the scenario where Y binds to the complex AX. The contrasting effects of the two mechanisms on the uptake of X in response to Y are shown in Figure 8.

The above mechanism essentially allows one component to lock another into the complex. Another view of protein complex formation comes from allostery. Here, it is possible for two inputs to act on one protein in a synergistic way, so that they are both in some sense locked in by one another, simply through the stabilization of a high affinity conformation. Examples in which this might be significant are widely available, and include the WASP family of proteins [1], A-kinase anchoring proteins [2], and phospholipase C [4]. This can be captured by a model of co-operative interaction between X and Y in binding A in which the preferential binding of the proteins to the active conformation shifts the population towards this active state. A simple representation of such a model is the following (see schematic in Figure 7d).(6)

Where *K_A_*_1 _and *K_A_*_2 _are association constants and *α *is the cooperativity constant between X and Y. Note that in the above model the same co-operativity constant appears in the last two expressions. This is done for simplicity, and ensures that the steady state of binding in the network corresponds to equilibrium conditions. Again, we see that signalling specificity may be increased through this mechanism. Just as in the previous cases, we find that the presence of Y increases the active output from pathway A but also inhibits pathway B (Figure 8).

It is worth pointing out that allosteric models of complex formation predict that this mechanism of crosstalk inhibition can occur bidirectionally, meaning that Y affects uptake of X and vice versa. This is in contrast to the sequential-binding model discussed above, where the component which binds first can affect uptake of the other, but not the other way round. It should also be noted that, while allosteric mechanisms are often proposed due to observed synergistic activation of a protein, the crosstalk inhibition effect identified here does not require such synergy. In the case considered here, if Y can activate A on its own, then allosteric effects may not appear to be significant. However, they can still allow cross-inhibition of pathways, and therefore signalling specificity.

Taken together we have seen how the presence of an extra element Y to the simple pathway coupling can lead to the activation of the relevant pathway and inhibition of the other (competing) pathway. Thus mechanisms of complex formation provide control settings to determine how pathways and processes may inhibit one another. Overall, additional elements can modulate the pathway structures in highly non-trivial ways, and this provides some insight into how additional elements in cellular signalling systems may act to modulate and control signal splitting between pathways.

### Coupling of switches

The basic model presented here made no assumptions about the nature of the pathways in which A and B are involved, and merely represented the binding of species to one another. However, in many cases proteins with multispecificity are also capable of enzymatically modifying the proteins with which they interact [5-9,11]. In this section we extend our model to examine some of the consequences which reversible multispecific posttranslational modification might have on the signalling properties of the system.

**Figure 9:**
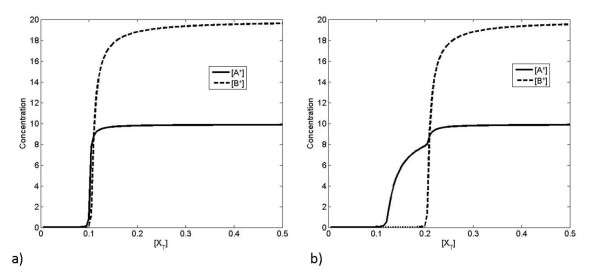
**Competition and specificity**. This figure shows how signalling specificity may be improved by competition effects. It considers the case of two switches which are regulated by the same upstream component. Each of the switches in isolation display switching behaviour at the same input dosage. a) shows the response of the switches in isolation, and b) shows the switches operating together. It is clear that it is not possible to choose input values for the isolated case which would result in mutual specificity. However, when they operate together mutual specificity can be obtained by having low inputs activating A and high inputs activating B, triggering the relevant switch.

**Figure 10:**
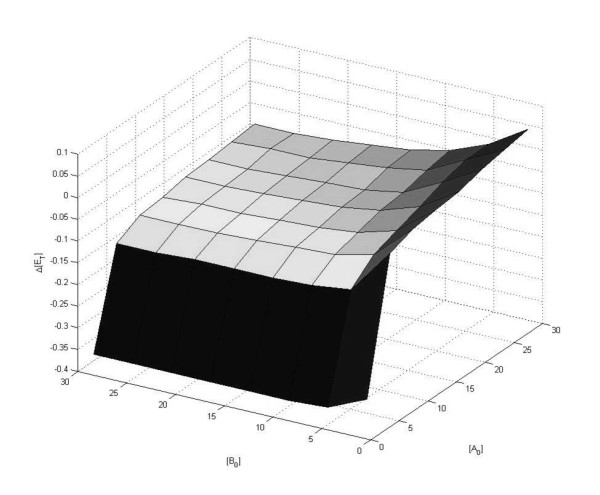
**Competition and timing**. This figure further examines the case presented in Figure 9 and shows how the difference in total enzyme ([X_T_]) concentrations at which the two switches become activated is kept consistent over a wide range of total concentrations of A and B. The difference plotted is the difference in input concentration between that where the A pathway is triggered and that where the B pathway is triggered.

We examine two generic cases of signalling through post-translational modifications - one involving only single reversible modifications of the substrates, and the other involving a double modification of one substrate. The first case is shown in Figure 9 and involves two switch-like pathways of Goldbeter-Koshland type [32], involving a single reversible posttranslational modification of each substrate (A and B) by the shared enzyme X. As previously, we begin by examining the steady state situation in terms of the conserved total quantities of X. The enzyme kinetics are chosen such that the affinity of X for A is higher than that for B, while keeping the overall steady state response of the switches in isolation very similar, as shown in Figure 9a. Now, when the two switches are connected via the common upstream component, we see that the high affinity pathway (A) is activated at a lower input level than the low affinity pathway (B). Thus the coupling of switches allows for a sequential activation in a well-defined order of the two pathways. In the model under consideration, we examined the effect of availability of A and B on the input dose difference between the activation of the switches, over a wide range of abundances of A and B (Figure 10). We note that this difference varies significantly when there are low concentrations of one component - this is the result of the uptake of X by the component being insufficient to couple to switches effectively. However, higher concentrations of A and B ensure suitable conditions for the coupling to occur effectively, and little difference is observed in their activation dosage-gap. Thus it is possible to have a sequenced activation of coupled switches arising from their activation through a common source.

Further to the steady state analysis, we can look at the effects of the system dynamics on signalling. As expected from the dynamic analysis of the response of coupled pathways to modulation of the shared component X, if the timescale of interaction between A and X (the high affinity interaction) is faster than the interaction between B and X, the dynamic pattern is similar to the steady state one - activation of A preceding that of B. Likewise, if the timescale of interaction between B and X is faster than the interaction between A and X, we get the reversed pattern, where at first B is active, followed by A. Again, adaptive behaviour in the low-affinity component, B, can result due if the faster pathway is the lower affinity pathway(results not shown).

**Figure 11:**
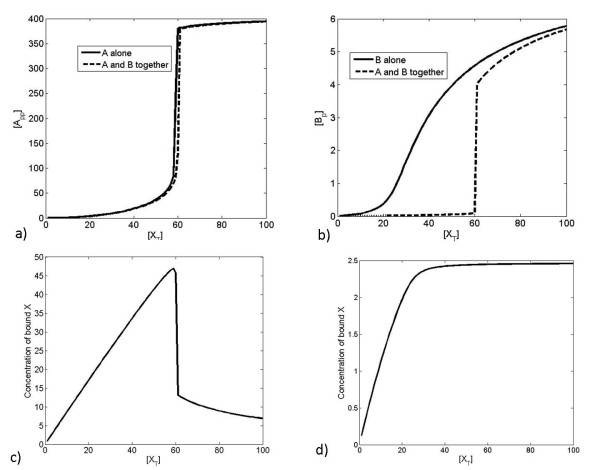
**Coordinated switching**. This figure illustrates another aspect related to the coupling of two switches through common upstream regulation. One of the switches (A) is a multiphosphorylation switch while the other (B) is a monophosphorylation switch. The response of the multiphosphorylation and monophosphorylation switches are shown in a) and b), respectively. The solid curves show their response to [*X_T_*] in isolation, while dashed curves show their response to [*X_T_*] when together. We see that the sharp switching of the multiphosphorylation switch can be "transmitted" to the monophosphorylation switch, resulting in coordinated switching at a well defined location. The reason for this is the sharp change in the uptake of enzyme by the multiphosphorylation switch, as the threshold is crossed, as shown in c), resulting in an increase in free enzyme. The response of the single phosphorylation switch does not exhibit this behaviour, as shown in d).

We now examine a different coupling of switches, of the enzyme with a single reversible modification of the substrate B, but two sequential reversible modifications of the substrate A (which we refer to as the multiphosphorylation switch), by the common enzyme X. The multiphosphorylation switch is capable of a range of behaviours - it is also capable of the switch-like monostable response of single phosphorylation [33], but is further capable of exhibiting bistability [34]. When exhibiting a monostable response, the behaviour of the combined system is as described above. However, when exhibiting a bistable response, a qualitative change in the behaviour is observed. The behaviour of the two switches in isolation and together is shown in Figure 11. We note that, when isolated from one another, pathway B displays only a slightly sigmoidal response, while pathway A displays a dramatic shift in response as a result of its bistability. In this case by coupling these two pathways via a common upstream regulator X, we see that as the upstream signal is increased past a threshold the multiphosphorylation switch is switched on. It is evident that at this point the switch involved in pathway A is also triggered, in fact in a more dramatic fashion than the regular switching of pathway A itself, and this is purely due to the coupling of the switches. The reason for this switching is seen in the shift in the available X observed when the bistability threshold is crossed (see Figure 11) - this shift is not observed when a monostable signalling threshold is crossed. Bistability in this case therefore results in significant qualitative changes to bidirectional signalling, as well as to the input-output response. Thus this example reveals that a strong switching behaviour in a pathway need not necessarily arise from characteristics embodied in that pathway, but may instead arise from coupling with other switches through shared components.

Taken together the examples show how it is possible to get sequential spaced switching from the coupling of switches with identical thresholds and also to get coordinated switching of pathways in a striking manner.

### Spatial signalling

This paper thus far has studied the coupling of pathways/processes through shared components and focussed exclusively on temporal signal processing. Many cellular processes involve aspects of spatial signal transduction, and the importance of spatial aspects in signalling is being increasingly recognized. In the context of our analysis, we systematically investigate phenomena introduced purely by differences in the diffusivity and localization of the components.

In order to do this, we assume that all components exist in a spatial domain. For specificity, we take this to be a 1-dimensional periodic domain, although most of the essential results remain valid in other domains. We now include the spatial element, by including spatial variation in one or more elements. Additionally, we examine the effects of one of the components being highly diffusible to see if this changes the effect of the interaction of the pathways in a non-trivial way. In this case, we will assume that A is highly diffusible, and likewise so is the complex AX. We emphasize that while we perform simulations and analysis in a 1-D periodic domain, our analysis and main conclusions are also relevant to other situations, for instance where X is initially present only in the membrane of the cell, while A is present, along with its complex, both in the membrane as well freely diffusible in the cytosol.

We will examine some scenarios which draw a direct contrast with the purely temporal signal processing in the case where the shared component, X, is modulated. We start by examining the situation where X is present only in part of the domain. A concrete example is if X is present at a non-zero level only in a specific region initially (for example having a square-pulse like spatial profile). No species is either produced or degraded. Now if all entities are non-diffusible then we expect that, in the region where X is present, at steady state there exists a balance between the concentrations of free X and complexes AX and BX, and this is determined exactly as above. Overall the conclusion therefore is that the complexes AX and BX are present only in the region where X is present, and the balance between these complexes is determined just as in previous sections.

Now we consider the effect of the high diffusivity of the pathway A. Analysis of the steady state equations reveals a number of points (see Appendix for details). Firstly, the total amount of A and B is constant at every location. Secondly, at steady state, at every location the binding/unbinding reactions between both A and B with X are at equilibrium. Thirdly, the complex AX attains an essentially spatially homogeneous profile. From this, it follows that at steady state both complexes AX and BX as well as X attain a homogeneous profile at steady state. The clear effect of the coupling of the pathways is seen, most directly in contrast to the case where both pathways were non-diffusible. Here the diffusion of one pathway has the effect of homogenizing the profile of both complexes. Thus we see that the regulation of the B pathway by X and the spatial profile of the complex BX is affected by the interaction with the diffusible A pathway.

**Figure 12:**
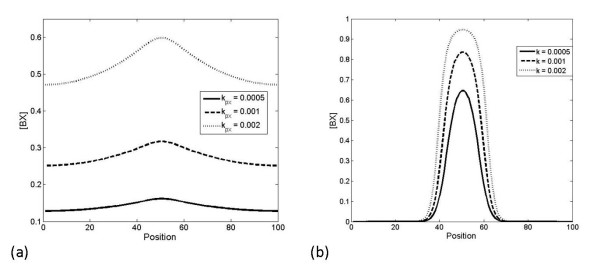
**Response to localized production of X**. This figure shows how the steady state spatial distribution of the complexes in response to varying rates of production of nondiffusible X. A and B are produced at a uniform rate across the domain, while X is produced in a localized region. a) shows the spatial distribution of BX as production of X changes, where both A and AX are diffusible. This allows BX to spread over the whole cell, even though it is not diffusible. The distribution of BX is almost uniform for low levels of production of X. b) shows the spatial distribution of BX as production of X changes, where A is the only diffusible component. Here a pronounced localization is observed. In both cases the AX profile is spatially uniform (not shown).

We now build on this to examine a related case where X is being actively produced in a inhomogeneous manner with a Gaussian like profile centred around the middle of the domain. All species are assumed to be degraded equally quickly. Analysis reveals again that the total A and B attain a uniform constant profile, that the complex AX attains a uniform profile, while both X and the complex BX are inhomogeneous. Increasing the production of X actually leads to an elevation in the level of BX at every spatial location, while only weakly reflecting the pronounced asymmetry in the spatial profile of X. This is shown in Figure 12. Complementary analytical results are performed in the Appendix.

In the above case, the activating signal was spatially inhomogeneous and this was the source of the spatial aspect of signalling. A slightly different case can be also examined, which fits naturally into our framework. This is the case where the activating signal is spatially homogeneous, but a localized sequestration reaction occurs. Thus in the above case we let the production of X be spatially homogeneous, but we regard the activation of the B pathway as occurring only locally in a restricted region. This can be described either by starting with homogeneous X in the domain, and B present only in a localized region, with no production or degradation of any species or alternatively by having homogeneous production of X (and A) and highly inhomogeneous production of B and having degradation of all species. Both situations provide essentially similar results. The first case is examined analytically in the Appendix.

We see that if both A and B are non-diffusible, the above sequestration effect of B would lead to AX being sharply depleted in this region, with AX showing a pronounced inhomogeneous profile and this being reflected in the profile of X as well. Further the balance between X, AX and BX can be determined exactly as performed in the purely temporal case.

Now, if the A pathway is highly diffusible, at steady state the AX profile becomes homogeneous and an analysis of the X profile reveals that it too becomes homogeneous. The BX profile reflects the pronounced heterogeneity, and now there is a global coupling between the levels of X and AX, and the profile of BX, which arises from a global conservation condition.

**Figure 13:**
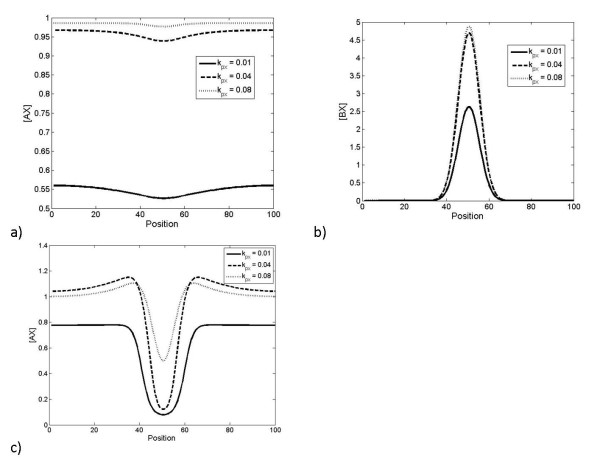
**Response of pathways when production of B is localized**. This figure shows how the steady state spatial distribution of the complexes AX and BX change in response to varying rates of production of nondiffusible X. A and X are produced at a uniform rate across the domain, while B is produced in a localized region (described by a sharp Gaussian profile centred around the middle of the domain). The spatial distribution of AX and BX as production of X changes is shown in a) and b), respectively. Localized production of B causes a very mild reduction in the production of AX around the centre and a sharp increase in BX there. Both A and AX are diffusible. In part c), only A is diffusible. Localized production of B causes a sharp reduction in the production of AX around the location at which B is produced.

Simulations describing this case are shown in Figure 13. In this case, if the X binding to B is strong, the net effect is a localized activation of B, and a consequently less strong regulation of A, which is nevertheless spatially homogeneous. It is also worth pointing out that for moderate levels of X production, the spatial profile of free X is itself close to uniform (and under these conditions are in agreement with analytical results performed for the case of no production/degradation of X) although as the rate of production is substantially increased the free X profile starts to reflect a dip in the region where B is present.

**Figure 14:**
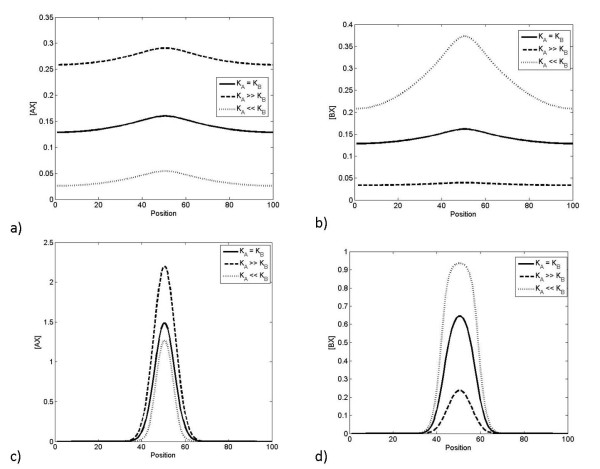
**Response when affinities are varied**. This figure shows how the steady state spatial distributions of the complexes AX and BX change with the relative affinities of A and B for X. In a) and b), both A and AX are the only diffusible components, while X is locally produced. In c) and d), only A is diffusible, while X is locally produced. It is seen that increasing the binding affinity of a particular complex increases its concentration, and that this effect is most significant for the nondiffusible complex, BX, which exhibits a more pronounced spatial variation.

Finally, we can consider the effects of differing affinities in this system. These effects are demonstrated in Figure 14, showing the response of AX and BX to a fixed, localised production of X, in the cases of A alone being diffusible, and A and AX both diffusing. In all cases, increasing the binding affinity of a complex increases its concentration globally, as expected. However, there is a difference in the behaviour of AX and BX to the difference in affinities - BX is more sensitive to changes in affinities than AX. The reason for this is that diffusion of A (or A and AX) allows the concentration of free A, and therefore the concentration of complexes, to remain relatively constant, while any uptake from a local pool of B cannot be compensated by the same mechanism. Therefore, AX is merely responding to changes in availability of X, while BX responds to changes in availability of X and B, and its sensitivity is consequently greater.

In the above, when we have considered the effects of a diffusible pathway, we have assumed that both A and AX are diffusible. One may also examine the case where A is the only diffusible component, and not AX. In this case, the response is similar to non-diffusible case, and AX exhibits a non-trivial spatial profile. The main difference when X is inhomogeneous arises in the fact that X is no longer limited by the local availability of A in contrast to the results in the purely temporal case.

Overall the above cases provide an illustration of the coupling of signalling pathways with shared components, where spatial aspects of signalling are important.

## Conclusions

This paper focussed on analyzing the interaction and coupling of pathways through shared components, a ubiquitous phenomenon in cellular networks. In this paper we examined this basic branching structure from a modelling and systems perspective.

We believe that a detailed systems analysis of signal processing in this setting is useful for multiple reasons. Firstly, it allows us to explicitly analyze the different features which affect signal processing, without being distracted by the details of a particular signalling context. Secondly, since such structures are repeatedly encountered biologically, it is only to be expected that different variations around this basic theme will be encountered, and the results here provide a platform and framework for analyzing these subsequently.

We developed a minimal modelling framework where we could examine the interaction of pathways with shared components. Since we include the possibility of production of all components, we were able to examine both dynamic and steady state responses to a variety of signals. More complex cases such as temporally regulated interacting pathways, with buffering of one pathway also form part of the framework. Each of the pathways of necessity interacted with the other, because of the shared component. These results were obtained using simulations and analytical work (see Table 1 for a summary of the main results).

We first examined the case where the common component is regulated by some external signal. Building on the work of Buchler et al [28,29], our studies reveal how, depending on the affinities of the common activating component to the two pathways, it is possible to obtain "ultrasensitivity" in the response of the component with a weaker affinity. We also showed how depending on the kinetic rates of binding/unbinding, the pathways could get activated in either temporal order or even concurrently. If there is a clear separation of affinities, and the low affinity pathway is the faster pathway, then for certain ranges of the input signal, the response of this pathway to a persistent stimulus is adaptive: this adaptation may be close to being exact if enough quantity of the high affinity component is present. A significant departure from the adaptive response is observed if the high affinity component is consumed. The saturation effect leading to inexact adaptation in this case is the consumption of the additional "inhibitory" high affinity component, and this is qualitatively similar to other saturating mechanisms leading to inexact adaptation (see the discussion in [35]).

In a similar manner we examined the case where the two other components, A and B, are regulated by external signals. Our framework allowed us to naturally apply and extend our analysis to this case too. We found that at steady state the high affinity pathway dominates. However, temporally, if the low affinity pathway was the faster pathway, then the response of this pathway was (for certain stimulus levels) partially adaptive (either underadaptive or overadaptive) and this was entirely due to the added high affinity component acting as an inhibitory component. If one regards the components A and B to be stimulated externally through some common source, then the signal transduction of the low affinity pathway in this regime is qualitatively similar to a feedforward adaptive signalling module.

Although our analysis was performed for the case of two pathways sharing a common component, the insights naturally generalize to the case where there are multiple pathways sharing a common component. Our results indicate that depending on the relative affinities, kinetics, and amounts of the individual components, different combinations of steady state responses (including possible "ultrasensitivity") and different kinds of temporal responses will be observed for the different complexes. This will be examined in detail subsequently. Our results have natural relevance for the (competitive) binding/activation of different entities by a common factor. Further, it is possible to predict the effect of modulating individual pathways here. Additionally, since many components are subject to temporal modulation (for instance, in concert with the progression with the cell cycle), this framework provides a natural platform for examining such effects systematically.

We then built on our basic analysis by examining additional factors built over the basic model structure (see Table 2 for a summary of models and findings). This is motivated by the fact that such additional elements modulating such pathways are naturally expected to be present in different ways in different contexts.

**Table 2:** Summary of results for variations on the basic model

Scenario	Result
An additional component is involved the uptake of X by one of the two pathways through a complex formation mechanism.	The additional component can control how signalling through X is divided between the two pathways. This may enhance signalling specificity. The degree of control the additional component has depends on the mechanism of complex formation.

The shared component is involved in two downstream pathways which display switching behaviour.	Where uptake of the shared component is significant, the switching behaviour of one pathway may influence that of the other. This can result in either a specific ordering of activation, or coordinated activation, of the two pathways.

One of the downstream components, and its complex with the shared component, is diffusible.	The shared component is spread across the domain. This results in uptake being significant across the domain, with the spatial distribution of the nondiffusible components also affected.

A summary of results for the biologically motivated variations on the basic model is presented. These demonstrate how the effects considered may play a role in diverse biological contexts.

In the first case we examined the effect of an additional component modulating one of the two pathways. It was shown that this could allow for greater specificity in signalling, effectively through inhibition of the one pathway by the other. Different modes of interaction via the extra component were considered, including sequential binding either before or after binding with the target, as well as co-operative binding to the target species. Analysis reveals that many of the relevant conclusions for all these cases were similar. This indicates how cellular systems may have naturally exploited their pre-existing set of molecules to add further layers of control and separation/differentiation between diverging pathways.

We additionally examined the effect of coupling of pathways which are involved nontrivial and highly non-linear signal processing. Thus we built on our existing modelling structure to include switch-like signalling in each pathway. Our analysis reveals that coupling two switches even with identical switching thresholds, can result in a well-defined order in the switching response, and further that under many conditions it is possible to maintain a robust "dosage gap" in the switching of the pathways. In other cases the interaction of two switches can lead to one switch being highly accentuated by coupling to a multiphosphorylation switch which is bistable. This is an example of co-ordinated switching in two pathways which arises from their coupling through shared components, and suggests that in some cases switch-like behaviour in some pathways can arise from their coupling to other pathways rather than their intrinsic switch-like behaviour.

Finally we expanded our model in a natural way to include spatial aspects in signalling and built on our early studies to examine signal processing in coupled pathways in spatial signal transduction. We showed that the coupling of a highly diffusible pathway to a non-diffusible pathway, could lead to effective redistribution, even of the non-diffusible complexes and hence provide a completely different spatial signalling profile. This reveals another facet of the coupling between pathways through shared components.

Our framework and analysis is relevant in a range of cellular settings. The activating of elements involved in controlling multiple pathways is observed in different settings, especially for proteins which interact promiscuously with a range of downstream targets (eg. Cyclin-dependent kinases [10] and ubiquitin ligases [12]). A special case is that of a protein which interacts with different isoforms of downstream proteins. One example of an effect similar to the response we have analyzed here occurs with anaphase promoting complex (APC)-mediated ubiquitination of cyclin A, securin and geminin: securin and geminin are ubiquitinated, and thus degraded, earlier than cyclin A [36]. Further, the presence of securin and geminin delays the ubiquitination of cyclin A. Other examples include the multiple GEFs (Guanine Exchange Factors) which target RhoGTPases. Our analysis of the is also relevant to competitive exclusive binding of multiple ligands to the same receptor, and is of special interest when the binding of each ligand triggers opposite responses (for e.g. CAMP and 8-CPT CAMP to CAR1 receptors in Dictyostelium [37])

In the case of the two separate components being modulated together, our framework provides insight into how different elements are targeted by multiple pathways, and how cells may have evolved strategies to reinforce or minimize the concurrence of signals. The presence of a host of additional proteins providing combinatorial control and selective tuning of individual pathways is a key aspect to be investigated to understand signal processing through classes of hub proteins, and is also likely to be highly relevant to selective targeting of pathways intended as drug targets. Our analysis is also relevant to the assessment of the deleterious effects of increased gene dosage (suggested to be the result of the promiscuous interaction of certain proteins, which is suppressed at low copy numbers) as well as their mitigation by selective degradation of unbound proteins which are also promiscuous [38-40].

The spatial aspect of signalling we have considered here is relevant to enzyme regulation of multiple components, all or some of which may shuttle between different compartments (for eg. membrane, cytosol, nucleus, ER) as well as the enzyme regulation of multiple components, some of which may be highly diffusible (eg. cGMP). Likewise the localized sequestration is observed when certain enzymes which are otherwise freely mobile, partially bind to anchor proteins at specific regions on the cell membrane or elsewhere in the cell. An example of this is the case of PKA which may be partially anchored in certain regions by suitable anchor proteins. Analogues of these spatial effects at the tissue level also exist. In developing Drosophila embryos it has been suggested that substrate competition for MAPK (itself present in a spatial pattern) coordinates the anterior and terminal patterning systems [6].

The presence of a common element between two pathways can serve to couple them. If the signal transduction in either or both pathways is highly non-linear, this can lead to a significant interference between the signal processing and distortion as a result. In which situations this actually happens due to the natural wiring of the cell, and under which conditions this is effectively minimized, is a topic which needs much more thorough investigation. It will be of interest to see how shared components may affect the interaction of other modules (e.g. [41]) and this is something which will be examined in the future.

Our results and insights have been obtained in a general setting, and thus we expect that many of these insights to be relevant of a wide range of systems. Using this framework, it is possible to build additional features like multiple-component signalling, combined converging and diverging signals as well as coupling of more complex downstream processes. Additionally, the analysis here provides insight into understanding to what extent control or temporal modulation of upstream signals may be propagated through multiple pathways and how this affects the manner in which pathways interact with one another. It is worth pointing out that the analysis here is relevant not only to natural signalling circuits but to synthetic circuits as well. In synthetic circuits a major challenge is to how a synthetically constructed circuit may interact with the host cell. One of the most basic interactions is the possibility of components in the synthetic circuit being also involved in other pathways in the host cell.

The network structure which embodies the diverging/converging pathways we have studied is ubiquitously observed. By examining the signal processing in this "splitter" or "converger" element from a systems perspective we can examine how such elements may be interfaced with other signal transduction elements both upstream and downstream. This will be invaluable both in understanding complex dynamics and control regulation and coupling of signalling in systems biology, but also be vital for starting to build synthetic circuits which usefully and optimally channelize signals for a range of purposes.

## Competing interests

The authors declare that they have no competing interests.

## Authors' contributions

Both authors contributed to the planning of the paper, the results which are presented here as well as the writing of the paper.

## Appendix: Analysis of models

In this section, we will analyze the models used in the text to extract additional insights which complement numerical simulations. As mentioned in the text, we have a common framework to account for the sharing of a component between two pathways or processes. This involves the binding of the common component X to two components A and B. In the most general case, the model includes the binding and unbinding reactions as well as the production of species X, A and B, as well as the degradation of all species. The equations describing this are given by(A1)

We begin as in the text, analysing the case where there is modulation of the shared component, X.

### 1.1 Modulation of the shared component

As mentioned in the text, the equation A1 includes production and degradation of X, A and B. We first restrict ourselves to the case where no production signal is directly affecting A or B. A production signal affects X and X affects both pathways A and B. Rather than vary every parameter in the model, we examine the model in a number of special cases, gradually increasing in complexity.

#### Case 1: There is no production or degradation of any entity

This is the simplest model in some respects. It is clear that in this case, the total amount of A (i.e. free and bound) is conserved and this is immediately seen by noting that d/dt([*A*] +[*AX*]) = 0. The total amount of A, denoted by *A_T _*is determined by the initial conditions. By exactly analogous reasoning, the total amount of B (free and bound) is a constant, denoted by *B_T_*. Finally there is a conservation for the total concentration of X and this follows by noting that d/dt([*X*]+[*AX*]+[*BX*]) = 0. The total amount of X is denoted by *X_T_*. Now at steady state, the following equations hold:(A2)

When [*AX*] and [*BX*] are eliminated from the above equations, the equation which results is a cubic equation, whose solution cannot be written explicitly, except for special parameter values. In some special cases, where X binds relatively weakly to A and B, the solution can be written down explicitly. In this case, we find that(A3)

The above solution is the limiting case of weak binding of X to A and B, resulting in a situation where (free) A and B are very far from depletion.

It is worth pointing out that if one of the species (e.g. B) satisfies the above condition, analytical solutions can again be obtained. Thus, if the binding of X to B was weak, this would imply that [*BX*] was essentially directly proportional to [*X*], and [*X*] could hence be written as a linear function of [*AX*], and this results in a quadratic equation for [*AX*] which can be solved explicitly.

#### Case 2: There is production and degradation of only X: the production of X serves as an input signal to the model

In this case, exactly as before the total amount of A (free and that in the complex AX) is conserved and likewise for B. Examining the total amount of X, we find that(A4)

We thus find that at steady state the total amount of free X is easily obtained as(A5)

From this and equation A1, it is an easy matter to find the steady state concentrations of the complexes as:(A6)

We see here that clearly the affinity of X for A affects how much A is present in a complex (and likewise for B). In this case we can obtain analytical expressions for the steady state concentrations of the complexes involved in the two pathways. However, in this special case, the level of free X is fixed at a constant value at steady state and this effectively decouples the two pathways. The conclusion therefore is that if X is being produced and degraded, the production/degradation of other species is necessary to achieve non-trivial coupling between the two pathways at steady state.

#### Case 3: The degradation rates of all species are equal (denoted by k_d_)

In this case since there is degradation of the A and B species, there must be some basal level of production. Now we see that(A7)

We immediately see from above that at steady state the total amount of A (free and complexed) is set at the level *A_T _*= *k_pa_/k_d_*, and likewise the total amount of B (free and complexed) is set at the level *B_T _*= *k_pb_/k_d _*and hence the total amounts of each of A and B at steady state may be regarded as conserved, as far as signalling from X is concerned.

We note in passing that while we have assumed all degradation rates to be equal, the above conclusion for the total amount of A actually only requires the degradation rates of free and complexed A to be equal (and likewise for B).

The total amount of X from above is also set at the value *X_T _*= *k_px_/k_d_*. The concentrations of the complexes can then be obtained from the equations(A8)

where *X_T_*, *A_T_*, and *B_T _*are as given above. The solution of these equations is formally identical to that of case 1, and the same conclusions hold good here as well.

##### "Ultrasensitivity" in the steady state response

As mentioned in the text, for our purposes "ultrasensitivity" involves heightened absolute and relative sensitivity to [*X_T_*] along with any concomitant threshold effects. The absolute sensitivity of a concentration to [*X_T_*] is defined as its derivative with respect to [*X_T_*], while the relative sensitivity is this quantity scaled by the ratio of the relevant concentrations. Here, we analyse the steady state response of the complete model in terms of the absolute and relative sensitivities to the total quantity of X, [*X_T_*]. In particular, we look at how these quantities behave in particular limits, when there is a significant difference in the affinities *K_A _*and *K_B_*.

We begin by deriving expressions for the absolute and relative sensitivities. Differentiating the steady state expressions given in equation 5 with respect to [*X_T_*] gives the absolute sensitivities:(A9)

The relative sensitivities are then given by:(A10)

From these expressions, we can explain the pattern in relative sensitivities shown in Figure 2b. First, when there is very little X present, very little complex is formed, and we have [*A*] ≈ [*A_T_*], [*B*] ≈ [*B_T_*], and [*X_T_*] << 1 (the amount of different complexes is proportional to the available X). At this point, noting that the derivatives in the above expressions are bounded, we simply have .

We can consider what happens at intermediate, albeit low, values of [*X_T_*] for the case of comparable [*A_T_*] and [*B_T_*], with *K_A _***>>***K_B _*>> 1. This is similar to the case considered in Figure 2. Here, at low levels of [*X_T_*], *K_A_*[*A*] ≫ *K_B_*[*B*] + 1, meaning that the absolute sensitivities are given by: *d*[*X*]/*d*[*X_T_*] ≈ 0, *d*[*AX*]/*d*[*X_T_*] ≈ 1, and *d*[*BX*]/*d*[*X_T_*] ≈ 0. Taking the relative sensitivities, as given by equation A10, and evaluating the derivatives (substituting - *d*[*AX*]/*d *[*X_T_*] for *d*[*A*]/*d*[*X_T_*] and - *d*[*BX*]/*d*[*X_T_*] for *d*[*B*]/*d*[*X_T_*], as given by the conservation conditions) gives:(A11)

Substituting the above approximations for the absolute sensitivities then gives:(A12)

This shows that, before A becomes saturated (i.e. while *K_A_*[*A*] ≫ *K_B_*[*B*] + 1 holds), the relative sensitivities of both [*X*] and [*BX*] to [*X_T_*] increase from 1, while that of [*AX*] decreases from 1. This is observed in simulations, as shown in Figure 2b.

In the limit of very high [*X_T_*], we can consider that [*X_T_*] ≈ [*X*], as the proportion of X taken up in complexes becomes small. Under these conditions, the equilibrium expressions in equation 5 can be rearranged to give:(A13)

where the numerators approach a constant value. This indicates the asymptotics for [*A*] and [*B*] as the total X becomes large (this can be justified carefully).

These expressions are small for high [*X_T_*], so we can consider that *K_A_*[*A*] + *K_B_*[*B*] ≪ 1, so 1 + *K_A_*[*A*] + *K_B_*[*B*] ≈ 1. Using this and A11 in equation A10 and evaluating the derivatives gives:(A14)

This result can also be obtained by incorporating the asymptotic expression for [*A*] and [*B*] for large [*X_T_*] and evaluating the relevant expressions.

This demonstrates what we expect intuitively - as [*X_T_*] becomes large, the relative sensitivity of free X asymptotes to one, while the relative sensitivities of the complexes AX and BX asymptote to zero.

##### Dynamic response

As in the text, the basic case examined (Case 1 above) reveals that at steady state X is taken up much more by the high affinity component, in the case where the affinities are very different. The discussion in the text showed that X is primarily taken up by the high affinity component A, and so at steady state the complex BX displays an ultrasensitive response as a function of total X in the system.

As mentioned in the text, this focuses only on the steady state, and the information (affinities) rely only on equilibrium constants rather than rate constants. If the individual rate constants of binding/unbinding to A are much faster than that of B, then dynamically too, if X is added, it is taken up by A first, before B.

Here we examine (in a setting analogous to case 1 above) the case that A is the high affinity component, but actually the slower pathway. To illustrate this we consider a case where the binding and unbinding rate constants for the interaction of X with A are changed to εk_a1_, εk_a2_, where ε << 1 is a small parameter. This ensures that the equilibrium constant remains the same.

Now for illustrative purposes we examine the situation where no X is present intially (only free A and free B are present). At time t = 0, an amount of X = [*X_T_*] is added. We see that just as in earlier cases [*A*] + [*AX*] = [*A_T_*] and [*B*] + [*BX*] = [*B_T_*] reflecting the conservation of A and B. Since the equations reflect a conservation of total X, it remains to solve two differential equations governing the concentrations of the complexes AX and BX:(A15)

Now we look at the first two expressions reveals that they are directly cast as fast-slow systems with time scale separation, and hence can easily be examined using singular perturbation techniques. We can immediately see that in the fast time scale [AX] is essentially zero, while [BX] builds up according to the binding/unbinding of X with B, as described by the second expression in equation A15, and eventually reaches a quasi-steady state where(A16)

This quasi-steady state can be obtained by solving the resulting quadratic equation, and reflects the uptake of X only by B. At a slower time scale, there is a very gradual "leaking" of X by its binding to A (described by the first differential equation (equation A15)), resulting in less X available to the B pathway since [*X*] + [*BX*] = [*X_T_*] - [*AX*], and during this long time scale, the X is gradually redistributed to A, with BX being in a quasi-equilibrium with B and free X, equation A16, at every instant. Eventually more X ends up being bound to A than B, reflecting the higher affinity of X for A. Note that this assumes that the amount of X added is not too high (in particular not in excess of A and B in total). If a very high amount of X is added, then both pathways will be eventually saturated.

### 1.2 Modulation of each pathway alone and together

We now examine our modelling framework for the case where A and B are each modulated alone and together. Here the signalling occurs through the production of A and B, which impinge upon X. The focus is to determine how the signals A and B affect the formation of the complexes AX and BX. We can consider some particular cases:

#### Case 1: The degradation rates of all species are identical

This is identical to Case 3 of the above subsection, except for the fact that the signals are transmitted through the production of A and B. The steady state analysis otherwise is identical to the above case.

#### Case 2: The only species being degraded are A and B

This is an exact analogue of case 2 in the previous section (there the only species being produced and degraded was X). Here an analysis of the equation A1 reveals that(A17)

The above immediately leads to the fact that the total X is conserved and equal to *X_T _*(determined from the initial condition). Further the steady state concentrations of A and B are set to *k_pa_/k_da _*and *k_pb_/k_db _*respectively. This provides the information needed to find the concentrations of the complexes:(A18)

### 1.3 Combinatorial effects in modulating pathways

In the text we examined how an additional element Y could significantly affect the signal processing through the coupled pathways. We now revisit this, and examine the effect of the additional element Y. We examined three cases in the text. Here, we will examine the effect of Y in two of the cases considered.

We noted from our studies that it was not easy to obtain analytical solutions for the coupled pathways, except in the case where X was the only species being externally produced and degraded. However, in this case, at steady state, the complex dynamics are decoupled. We will revisit the basic case where no species is being produced or degraded.

In the first case, X binds not directly to A but to the complex AY. The binding of A to Y results in the complex AY. Assuming, for simplicity, an excess of Y, we have the concentrations of the complexes AY and AYX:(A19)

We have, through an abuse of notation used the same binding unbinding constants as in previous cases. Since it is only AY which binds to X, the conservation of A leads to the condition(A20)

This means that(A21)

As mentioned in the above sections, for the basic model, explicit analytical results are obtained only in the case of weak coupling. Now, the steady state for X, as obtained above is simply(A22)

with [*AY*] obtained from above. The point is that the concentration of [*AY*] depends on the concentration of Y, and clearly with no Y there is no complex. On the other hand increasing the Y increases the concentration of AY and this acts to inhibit the concentration of BX. This complements the numerical simulation, showing the same effect.

In the second case we have the situation of Y binding to AX, with an equilibrium constant which we will again refer to as k_ay_. In this scenario, X can bind to A and B, but Y binds to the complex AX to give AXY. The analysis of this case follows from our analysis of the simple coupled pathways. Again, for simplicity, we will assume that there is no production or degradation of any quantity. This immediately leads to the conservation of total X, total A and total B (free and complexed). For simplicity we will assume that Y is in excess.

Just as before, some basic analytical results are obtained in the case where the complexed A and B have concentrations which are small relative to their total amounts. From the above equations, we have(A23)

Thus, we have the total amount of active A and B respectively are given by(A24)

This equation again simply reveals that increasing the concentration of Y tends to increase the concentration of X taken up by A at steady state and decrease the concentration of X taken up by B at steady state. This points to that fact that increasing Y can lead to the inhibition of the B pathway at steady state. Similar conclusions continue to hold good if the complexed A and/or B are substantial fractions of their respective amounts, except that explicit close-form solutions are difficult to obtain.

The case of co-operative binding can again be analyzed in a similar manner, and we do not repeat the analysis here.

### 1.4 Spatial signalling

In this subsection, we will analyze the coupled pathway structure to include spatial effects. We will analyze the resulting model in a manner which builds on, and complements, the above analysis. As mentioned in the text, A will be assumed to be the diffusible pathway. Both A and AX are assumed to be highly diffusible. The model is formulated on a 1-D periodic domain for simplicity. Thus to start with we will consider the following situation.

#### Case 1: No production or degradation of any species

X can form complexes with A and B. X is initially present only in a localized region while A and B are present everywhere in the spatial domain. At the outset we note that if A and AX are non-diffusible, this exactly reduces to the case of coupled pathways analyzed earlier and the net result would be an activation of both pathways in the region where X is present. The total amount of X at any location is fixed by the initial condition, and from the equations presented in the earlier subsection (based on the model in equation A1), we see that this affects the steady state concentrations of AX and BX. We also note that if no X is present in particular regions, the concentration of AX and BX are both zero.

We now examine the case where the pathway A is highly diffusible. Assuming both A and AX have the same diffusion coefficient (*k_da_*) and adding the equations for A and AX we have(A25)

This shows that the total amount of A at any location satisfies the diffusion equation, and in particular if the total amount of A is constant at every location initially, then this remains so in the subsequent dynamics. Thus we have [*A*] + [*AX*] = *A_T _*at every location, exactly as before.

Now at steady state for BX, we have a balance between the binding and unbinding reactions just as before(A26)

This is exactly as in the earlier case. Now examining the steady state for AX results in(A27)

We see that, in this equation, as *k_d _*becomes large, the steady state profile of AX approaches a flat profile. This is easily verified by performing a regular perturbation analysis in the small parameter 1/*k_d _*as has been performed elsewhere.

Now since the steady state concentration profile of AX is constant (flat), and since we have a conservation of A, from above, it also follows that the steady state concentration profile of free A is also constant.

By examining the steady state for X, which is consumed in complexes with A and B, we see that the steady state for X necessarily implies an equilibrium between binding and unbinding to A. In other words, we have:(A28)

This is because there is a local equilibrium in the binding/unbinding to B at steady state, and the only contributor in the dynamics is the binding and unbinding to A. Now from above since A and AX have constant steady state profiles, it immediately follows that the steady state profile of X from the previous equation is also constant.

Thus in summary we have established that even though X is present only locally in certain regions, at steady state the spatial profiles of A, X, B, AX and BX are all constant. They can thus be solved exactly as in the purely temporal case, with the only modification being that the total amount of X at any location (which is constant) is determined from a global condition:(A29)

Here 2π is the length of the domain. This determines the total X at any location at steady state. Note that the above assumes that initially X is present only in an uncomplexed form. If it is present partially in complexed forms, then these have to be included in the integral.

Overall the significance of the above analysis is that owing to the diffusivity of the pathway A, at steady state an equidistribution of all species across the domain occurs.

We now examine some other related cases.

#### Case 2: A uniform amount of X is present initially (and likewise A). X is sequestered by B in a localized region

The sequestration of X by B is modelled by having B present initially in only a localized region. In general the total B need not necessarily be constant in this region.

In the above case we see that if A and AX are non-diffusible, we simply note that outside the region where B is present X and AX are uniform (determined simply by the binding and unbinding of A and X). In the sequestration region the balance between X, AX and BX would be described precisely by the purely temporal model.

We now examine the case when A and AX are highly diffusible. We see that much of the analysis of Case 1 remains relevant. There is a conservation of B so that *B *+ *BX *= *B_T_*, where the total B is now a function of position.

A steady state for complex BX implies that the binding unbinding of B and X is in equilibrium, just as before. Just as in case 1 the total amount of free and complexed A is a constant, set equal to *A_T_*. Again owing to the high diffusivity of AX, it attains a spatially uniform steady state profile and this is also the case for A. Further, from the steady state of X, we see that X must be in local equilibrium with A and hence uniform as well. This indicates the BX is determined by:(A30)

Thus overall we see that the high diffusivity of AX implies a uniform profile of AX and X, but a localized profile of BX in a manner proportional to the total amount of B present at that location. The uniform levels of X and AX are of course coupled to the BX profile through a global conservation law. The main point, however, is that the localized sequestration of X by B does not lead to a localized dip in free X when AX is highly diffusible.

Note that the above conclusion is also true if an inhomogeneous B is created through an inhomogeneous production of B.

#### Case 3: X is produced in an inhomogeneous manner, and all species are degraded at the same rate

Here we examine the slightly more complicated analogue of Case 1. Here all species are produced and degraded. Adding the equations for A and AX, we have(A31)

Now, at steady state we see that the total amount of A (which equals [*A*] + [*AX*]) is given by(A32)

Similarly the total amount of B is fixed at every location at a value *k_pb_/k_d_*.

Now owing to the high diffusivity of AX, it attains a spatially uniform profile (and hence so does A). However, in this case there is an inhomogeneous production of X which ensures that the spatial profile of X is not uniform. By using the steady state conditions for BX in the equation for X, we see that the steady state profile of X is governed by the equation:(A33)

Now in this equation, we note that the profiles of AX and A are uniform. The only source of explicit heterogeneity arises for the production of X. In fact if the fraction of B in the complexed form BX is small the above equation reduces to a linear equation for X, where the "production" terms involve dissociation from AX, which is spatially uniform, and the production which is spatially non-uniform. However, the concentrations of AX, BX and X are coupled through a global conservation condition. We also note that when the production rate of X, the situation reduces to the cases analyzed previously.

Overall the analysis provides direct insight into the source of the heterogeneity of the X profile (and hence the BX profile).

#### Case 4: Only free A diffusible

In this subsection we very briefly examine a variation of the above analysis where the free species A is diffusible but the complex is non-diffusible.

If we examine the first case above where X is present inhomogeneously initially, we see from the equations at steady state that A attains a uniform profile. BX is in local equilibrium with X and so is AX. Thus in this case we have a situation where BX and AX are present only where X is present and in local equilibrium with X, and the equations are formally similar to the purely temporal signalling case. However the level of A is determined by a global conservation condition, and depends on the amount of A present as a complex.

In the other case, where a localized sequestration reaction involving B occurs, again at steady state free B in equilibrium with BX and at steady state A has a uniform profile. In this case however, AX is locally depleted in the sequestration region, and the (uniform) level of A reflects the amount of A present as a complex in this region via a global conservation condition.

## Supplementary Material

Additional file 1 **Additional modelling and parameter values**. The mathematical models of single and double reversible phosphorylation switches are described, and the parameter values used to obtain all figures, are given. Click here for file
