# Supplementary material for: The coupling of pathways and processes through shared components
Source: BMC Syst Biol. 2011 Jun 29;5:103. doi: 10.1186/1752-0509-5-103 (PMC3162518; doi:10.1186/1752-0509-5-103)
Supplement: Additional file 1 — Additional modelling and parameter values. The mathematical models of single and double reversible phosphorylation switches are described, and the parameter values used to obtain all figures, are given. [file 1752-0509-5-103-S1.DOCX]

# Additional modelling and parameter values

## Model of single reversible phosphorylation

A single reversible phosphorylation, with uptake of the kinase, can be described by combining a mass-action based phosphorylation reaction with a Michaelis-Menten type dephosphorylation reaction. This gives the equations:

$$\frac{d[E]}{dt}=v_{2}+v_{3}-v_{1}$$

$$\frac{d\left[ AE \right]}{dt}=v_{1}-v_{2}-v_{3}$$

$$\frac{d\left[ A_{p} \right]}{dt}=v_{3}-v_{4}$$

$$\frac{d[A]}{dt}=v_{2}+v_{4}-v_{1}$$

Where:

$${v_{1}=k}_{a1}\left[ A \right][E]$$

$${v_{2}=k}_{a2}\left[ AE \right]$$

$${v_{3}=k}_{a3}\left[ AE \right]$$

$$v_{4}=\frac{k_{a4}\left[ A_{p} \right]}{K_{ma4}+\left[ A_{p} \right]}$$

Similar equations are obtained for the phosphorylation of B.

## Model of double phosphorylation

We use the model given by Markevich *et al* [1]. In the notation used here, they are:

$$\frac{d[E]}{dt}=u_{2}-u_{1}+u_{4}-u_{3}$$

$$\frac{d[F]}{dt}=w_{3}-w_{1}+w_{6}-w_{4}$$

$$\frac{d[A]}{dt}=w_{6}-u_{1}$$

$$\frac{d[A_{p}]}{dt}=u_{2}-u_{3}+w_{3}-w_{4}$$

$$\frac{d[A_{pp}]}{dt}=u_{4}-w_{1}$$

$$\frac{d[AE]}{dt}=u_{1}-u_{2}$$

$$\frac{d[A_{p}E]}{dt}=u_{3}-u_{4}$$

$$\frac{d[A_{pp}F]}{dt}=w_{1}-w_{2}$$

$$\frac{d[A_{p}F]}{dt}=w_{2}-w_{3}$$

$$\frac{d[{A_{p}F}^{*}]}{dt}=w_{4}-w_{5}$$

$$\frac{d[AF]}{dt}=w_{5}-w_{6}$$

Where:

$${u_{1}=k}_{1}\left[ A \right]\left[ E \right]-k_{-1}\left[ AE \right]$$

$${u_{2}=k}_{2}[AE]$$

$${u_{3}=k}_{3}\left[ A_{p} \right]\left[ E \right]-k_{-3}\left[ A_{p}E \right]$$

$${u_{4}=k}_{4}\left[ A_{p}E \right]$$

$${w_{1}=h}_{1}\left[ A_{pp} \right]\left[ F \right]-h_{-1}\left[ A_{pp}F \right]$$

$${w_{2}=h}_{2}\left[ A_{pp}F \right]$$

$${w_{3}=h}_{3}\left[ A_{p}F \right]-h_{-3}\left[ A_{p} \right][F]$$

$${w_{4}=h}_{4}\left[ A_{p} \right][F]-h_{-4}\left[ {A_{p}F}^{*} \right]$$

$${w_{5}=h}_{5}\left[ {A_{p}F}^{*} \right]$$

$${w_{6}=h}_{6}\left[ AF \right]-h_{-6}\left[ A \right][F]$$

## Parameter values

We provide parameter values for all simulations shown in the figures.

### Figure 2

Total amounts of A and B were maintained ([*A_T_*] = [*B_T_*] = 1), and the simulations were run to steady state. The affinities used were *K_A_* = 10^3^, *K_B_* = 70.

### Figure 3

*K_A_* = 10^3^, *K_B_* = 70. All degradation constants were equal: *k_dx_* = *k_da_* = *k_db_* = *k_dax_* = *k_dbx_* = 0.01. Production of A and B was set at the same rate: *k_pa_* = *k_pb_* = 0.02. Initially, no X is produced (*k_px_* = 0), but at the start of the simulation it is stepped up to *k_px_* = 0.08. In a) the timescales of both interactions, set by the disassociation rates, are equal: *k*_a2_ = 1/*τ_A_* = *k*_b2_ = 1/*τ_B_* = 1. In b), *k*_a2_ = 1/ *τ_A_* = 1, *k*_b2_= 1/ *τ_B_* = 0.001. In c), *k*_a2_ = 1/*τ_A_* = 0.001, *k*_b2_= 1/*τ_B_* = 1. In d) *k*_a2_ = 1/*τ_A_* = 0.001, *k*_b2_= 1/*τ_B_* = 1, with 0.5 X being added at t=0.

### Figure 4

The affinities here are equal, *K_A_* = *K_B_* = 1, while the timescales differ: *k*_a2_ = 1/ *τ_A_* = 1000, *k*_b2_= 1/ *τ_B_* = 0.1. The production and degradation of all species are initially equal: *k_dx_* = *k_da_* = *k_db_* = *k_dax_* = *k_dbx_* = *k_px_* = *k_pa_* = *k_pb_* = 0.01. The production rate of X is then pulsed up to a peak of 0.11 over a time of 1 (arbitrary units).

### Figure 5

The affinities used were *K_A_* = 10^3^, *K_B_* = 70. All degradation constants were equal: *k_dx_* = *k_da_* = *k_db_* = *k_dax_* = *k_dbx_* = 0.01. Production of X was the same: *k_px_* = 0.01. In a), production of A was stepped up from *k_pa_* = 0 to *k_pa_* = 0.02 at *t* = 0, while production of B was maintained at *k_pb_* = 0.02. In b), production of B was stepped up from *k_pb_* = 0 to *k_pb_* = 0.02 at *t* = 0, while production of A was maintained at *k_pa_* = 0.02.

### Figure 6

The parameters were identical as those for figure 5, except that here the production of both A and B was stepped up from *k_pa_* = *k_pb_* = 0 to *k_pa_* = *k_pb_* = 0.02 at *t* = 0.

### Figure 8

In part a), *K_A1_* = 5, *K_A2_* = 20, *K_B_* = 10. In part b), *K_A1_* = 20, *K_A2_* = 20, *K_B_* = 10. In c), just A, X, and Y were considered, and *K_A1_* = 20, *K_A2_* = 20 was taken for both mechanisms, allowing comparisons to be drawn. In d), *K_A1_* = 1, *K_A2_* = 1, *K_B_* = 10, *α* = 100.

### Figures 9 and 10

Here, the kinetics of the two switches differ only in the rate of association between enzyme and substrate. The complete parameters are: *k_a1_* = 100, *k_a2_* = 1, *k_a3_* = 10, *k_a4_* = 1, *K_ma4_* = 0.01, *k_b1_* = 10, *k_b2_* = 1, *k_b3_* = 10, *k_b4_* = 1, *K_mb4_* = 0.01. There is no production and degradation of proteins involved, since we look at the steady state response.

### Figure 11

The parameters used for the multiphosphorylation (activating A) are identical to those given in Markevich *et al* [1]. These are: *k_1_* = 0.02, *k_-1_* = 1, *k_2_* = 0.01, *k_3_* = 0.032, *k_-3_* = 1, *k_4_* = 15, *h_1_* = 0.045, *h_-1_* = , *h_2_* = 0.092, *h_3_* = 1, *h-_3_* = 0.01, *h_4_* = 0.01, *h_-4_* = 1, *h_5_* = 0.5, *h_6_* = 0.086, *h_-6_* = 0.0011. The parameters for the monophosphorylation (activating B) are *k_b1_* = 0.02, *k_b2_* = 1, *k_b3_* = 0.4, *k_b4_* = 1, *K_mb4_* = 0.1.

### Figures 12 and 13

Here, the interactions are all equal affinities and timescales, *K_A_* = *K_B_* = 70. The degradation rates for all components, and production rates of A and B, are equal: *k_dx_* = *k_da_* = *k_db_* = *k_dax_* = *k_dbx_* = *k_pa_* = *k_pb_* = 0.01. Non diffusible components had *D* = 0, while diffusible components had *D* = 100. Production of X was varied as indicated. A Gaussian production term was used for localised production, with a standard deviation of 5% of the domain size.

### Figure 14

Here, the conditions are as in figures 12 and 13, except for the affinities, which are varied. Here the low affinity component had *K* = 5, while the high affinity component had *K* = 50. Where affinities are equal, they are each set to *K* = 5. Production of X was maintained constant at, *k_px_* = 0.001.

1. Markevich, N.I., J.B. Hoek, and B.N. Kholodenko, *Signaling switches and bistability arising from multisite phosphorylation in protein kinase cascades.* J Cell Biol, 2004. **164**(3): p. 353-9.
